# Our Journal

**Published:** 1880-07-20

**Authors:** 


### *OUR JOURNAL.*

The present number of our Journal will visit many medical men not subscribers. We respectfully ask them to consider the claims of this Journal to their support. For ten years the editors have labored hard and expended much to establish a practical and useful Medical Journal in the interest of the profession, and as the organ of the busy practitioners throughout the country. The size and plan of the JOURNAL are such as experience has shown to be the best for the majority of medical men, furnishing in a compact form the largest possible amount of information for the least possible amount of cost. We have secured a large list of subscribers, and have received from them abundant evidence of approval as to the conduct and practical usefulness of our Journal. Yet there are thousands of medical men who do not take a Journal, and who seem to feel no interest in developing the medical literature of their section, or in keeping pace with the progress of their profession. There are duties which we owe to ourselves as true and conscientious men, and to our patrons, who have a right to expect the practitioner to know and to give them the benefit of the latest and best that is known to the profession.

Every true and honorable physician must see and acknowledge the truth of what we have said on this point. Every practitioner should take and read at least one good Medical Journal; and if only one, let that be one in his own section, as duty begins at home. And he should not only take it, but contribute as far as he may be able to its pages any useful and practical information which may be discovered or developed in his experience as a practitioner.

Without further argument upon what must be manifest to every reflecting and intelligent practitioner touching his duty in this matter, we kindly and earnestly appeal to every one who reads this article to subscribe for our Journal, and unite with us in our efforts to sustain a useful and good enterprise, to benefit the profession and to push forward the car of medical progress in our section. Send on your names for 6 or 12 months, payable on reception of the first number. The volume begins with January number, but may commence at any time. See club rates, terms, &c. on title page of JOURNAL.

